# Food-Based Antioxidant Nutrition for Exercise Recovery and Training Adaptation: A Narrative Review and Conceptual Framework for Redox Signaling, Dietary Matrices, and Periodized Application

**DOI:** 10.3390/nu18132115

**Published:** 2026-06-29

**Authors:** Hua Yang, Jingmei Dong, Jing Yang, Chieh-Chen Wu, Chun-Hsien Su

**Affiliations:** 1 College of Physical Education and Health, Gansu Minzu Normal University, Hezuo 747000, China; 0600021@gnun.edu.cn; 2 Department of Physical Education, Tongji University, Shanghai 200092, China; djm1969@tongji.edu.cn; 3 International Center for Health Information Technology (ICHIT), College of Medical Science and Technology, Taipei Medical University, New Taipei 23564, Taiwan; d610103003@tmu.edu.tw; 4 Department of Physical Education, Putian University, Putian 351100, China; 5 Department of Exercise and Health Promotion, Chinese Culture University, Taipei 111369, Taiwan

**Keywords:** polyphenol-rich foods, dietary nitrate, tart cherry, beetroot, food matrix, gut microbiota, oxidative stress, inflammation, endothelial function, exercise metabolism

## Abstract

Exercise-induced reactive oxygen and nitrogen species (RONS) serve as crucial signaling molecules for training adaptation, mitochondrial biogenesis, and inflammatory resolution, rather than being mere markers of oxidative damage. Chronic or excessive high-dose antioxidant supplementation may suppress these vital redox-sensitive pathways. Consequently, this narrative review examines food-based antioxidant strategies as approaches for redox modulation, meaning support for recovery and redox homeostasis without indiscriminately suppressing exercise-induced redox signals that may contribute to training adaptation, while emphasizing the distinction between whole-food matrices and isolated supplements. A structured literature search was conducted across major electronic databases, including PubMed, Web of Science, Scopus, and SPORTDiscus. The search focused on intersecting themes of exercise physiology, redox biology, and sports nutrition. The reviewed evidence includes short-term human intervention studies, systematic reviews, meta-analyses, and mechanistic studies examining tart cherry, berries, pomegranate, cocoa, green tea, beetroot, extra virgin olive oil, and Mediterranean-style dietary patterns. Overall, the evidence suggests that these food-based strategies may influence recovery-related outcomes through mechanisms extending beyond direct radical scavenging, including inflammatory regulation, vascular function, and gut-derived metabolism; however, the strength and consistency of findings vary by food source, outcome, dose, timing, study population, dietary matrix, and bioavailability. Current literature does not support universal, fixed daily antioxidant use. Food-based strategies appear most appropriate during periods of elevated recovery demands, such as heavy training blocks, congested competition, muscle damage, or environmental stress. Food-based antioxidant nutrition should therefore be interpreted as a conceptual, evidence-informed approach to periodized and context-specific recovery support, rather than as a universal or evidence-graded guideline, because much of the available evidence derives from short-term and heterogeneous intervention studies. These strategies should complement foundational sports nutrition practices (energy availability, macronutrient distribution, hydration, and sleep) when balancing the preservation of long-term training adaptations with the need for acute recovery.

## 1. Introduction

Exercise training increases metabolic demands on skeletal muscle, the cardiovascular system, and other energy-regulating tissues. Mitochondrial electron transport accelerates along with the increased oxygen demand, and some enzymatic and inflammatory pathways become more active. These processes generate reactive oxygen species and reactive nitrogen species [1,2,3,4,5], which are frequently discussed in relation to oxidative stress, nitrosative stress, muscle fatigue, inflammation, and post-exercise tissue recovery. For decades, this response to redox stimuli was mostly seen as an unfortunate side effect of high or prolonged activity. This interpretation was understandable in contexts where excessive redox disturbance was associated with lipid peroxidation, protein oxidation, muscle damage, delayed recovery, and performance impairment [1,2,6,7,8]. However, it is now too limited to treat exercise-induced redox activity merely as damage. At the same time, the interpretation of exercise-induced redox activity remains debated, particularly with regard to when antioxidant exposure supports recovery, produces neutral effects, or interferes with redox-sensitive training adaptations. In addition, ongoing training drives physiological adaptation [3,4,9] with redox-sensitive signaling.

Thus, reactive oxygen and nitrogen species create a central dilemma for exercise nutrition. At moderate and transient concentrations, these molecules act as signaling mediators rather than simply toxic by-products. They activate pathways implicated in mitochondrial biogenesis, endogenous antioxidant defense, vascular regulation, inflammatory resolution, and skeletal muscle remodeling [3,4,5,9]. Nuclear factor erythroid 2-related factor 2 (Nrf2), AMP-activated protein kinase (AMPK), peroxisome proliferator-activated receptor gamma coactivator 1-alpha (PGC-1α), and nitric oxide (NO)-related signaling are among the redox-sensitive mechanisms that may explain why a certain degree of redox stress is necessary for exercise adaptation [10,11,12,13]. The training goal is not to remove oxidative and nitrosative stress. It is to produce a stimulus sufficiently large to enable adaptation but recoverable nonetheless. When this response becomes excessive, prolonged, or mismatched with recovery capacity, the same biological network may shift toward maladaptation, chronic inflammation, fatigue, and reduced training quality [1,2,6,7,8]. Antioxidant nutrition must therefore be considered within a narrow range between supporting recovery and preserving beneficial redox signaling.

This explains why antioxidant use remains debated in sports nutrition. Antioxidants may help reduce oxidative damage, inflammation, and soreness after exercise, especially during heavy training, congested competition, environmental stress, or periods of inadequate dietary intake [6,7,8,14]. However, some reports suggest that long-term or high-dose use of isolated antioxidants such as vitamin C and vitamin E may attenuate several training adaptations, for example, mitochondrial biogenesis, insulin sensitivity, and endogenous antioxidant enzyme responses [14,15,16,17]. Current evidence does not support the simple assumption that antioxidant supplementation is always harmful. However, it does challenge the traditional “more is better” assumption. Dose, timing, chemical form, training phase, baseline diet, and target outcome all influence interpretation. This distinction is especially relevant because recreationally active individuals may have different recovery needs, dietary baselines, and adaptive targets from highly trained athletes exposed to high training loads, travel, and congested competition schedules [6,7,8,14,15,16,17]. A strategy that supports short-term recovery during competition may not have the same relevance during a training block designed to maximize mitochondrial or muscular adaptation, although recovery support and adaptation are not always opposing outcomes.

Food-based antioxidant approaches require a different interpretation from high-dose isolated supplementation. The key question is not whether antioxidant-rich foods can reduce oxidative stress in general, but whether they can modulate redox load without suppressing cellular signals required for training adaptation. Findings from capsule-based antioxidant trials cannot be directly extrapolated to whole foods or dietary patterns. In this review, the food matrix refers to the physical and chemical structure of whole foods and the way bioactive compounds are packaged together with polyphenols, vitamins, minerals, fiber, nitrate, essential fatty acids, amino acids, and other constituents that may interact biologically [18,19]. This matrix can influence digestion, absorption, gut microbial metabolism, endothelial function, immune modulation, and exercise-related signaling in ways that differ from single-nutrient supplementation [18,19]. Polyphenol-rich foods such as berries, tart cherry, pomegranate, cocoa, green tea, and extra virgin olive oil are better understood as potential modulators of redox and inflammatory responses than as simple radical scavengers [18,19]. Nitrate-rich vegetables such as beetroot and leafy greens further extend this model by linking dietary strategies with nitric oxide bioavailability, vascular function, and exercise performance [12,13]. Although several recent reviews have discussed overlapping mechanisms among antioxidant supplementation, polyphenol-rich foods, nitrate intake, recovery, rehabilitation, and training adaptation, practical integration across food matrices, timing, recovery demands, and training objectives remains less fully developed. This remaining translational gap limits practical interpretation for athletes and exercise professionals who must balance short-term recovery with long-term adaptive signaling.

This review investigates food-based antioxidant nutrition as an issue of timing, matrix, and training purpose. It explores how reactive oxygen and nitrogen species from exercise can be adaptive signals under certain conditions and as causes for damage or delayed recovery under others. The article also distinguishes high-dose isolated supplementation of antioxidants from those approaches delivered through whole foods, beverages, meals, and dietary patterns. Berries, tart cherry, cocoa, pomegranate, green tea, beetroot and other nitrate-rich vegetables, extra virgin olive oil, and Mediterranean-style dietary patterns are considered in relation to food matrix, dose, timing, recovery demand, baseline diet quality, and individual responsiveness. The goal is to develop a context-specific model for determining when food-based interventions may be useful, how they can be adapted to recovery demands, and how they may be integrated with training objectives. To improve the focus of this narrative synthesis, the review was guided by three questions: (1) How do exercise-induced redox responses contribute to both recovery challenges and training adaptations? (2) How do food-based antioxidant strategies differ from high-dose isolated antioxidant supplementation in terms of dietary matrix, bioavailability, and physiological targets? (3) Under what training or competition conditions may food-based antioxidant strategies be most appropriately periodized to support recovery without unnecessarily suppressing adaptive signaling?

**Figure 1:**
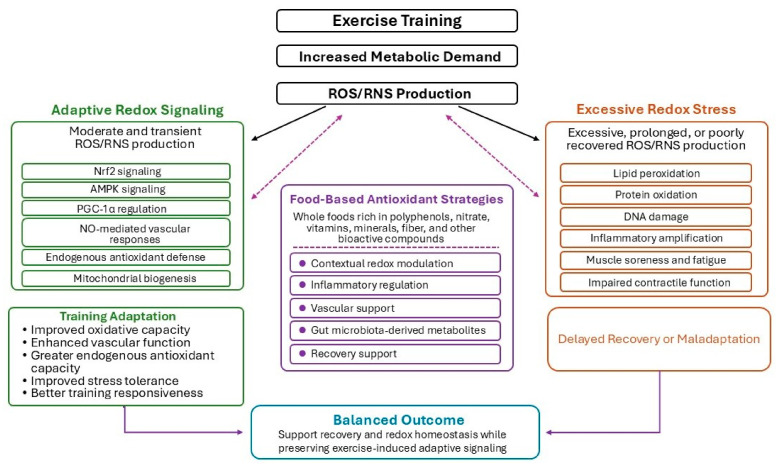
A conceptual framework connecting exercise-induced redox signaling, food-based antioxidant strategies, recovery, and training adaptation. Exercise training raises metabolic demand and induces ROS/RNS generation. Moderate and temporary ROS/RNS responses may act as adaptive signals by mediating Nrf2 activation, AMPK signaling, PGC-1α regulation, NO-mediated vascular responses, endogenous antioxidant defense, and mitochondrial biogenesis. Through these pathways, redox signaling may influence oxidative capacity, vascular function, stress tolerance, and long-term training adaptation. When ROS/RNS production is excessive, prolonged, or poorly resolved, however, redox stress can also promote lipid peroxidation, protein oxidation, DNA damage, inflammatory amplification, muscle soreness, fatigue, impaired contractile function, and delayed recovery. Food-based antioxidant strategies are proposed as approaches that deliver redox-active compounds within whole-food and dietary-pattern matrices. Solid arrows indicate proposed directional relationships, whereas dashed arrows indicate indirect or context-dependent relationships. They do not suppress exercise-driven redox signaling in an indiscriminate manner, but rather support recovery and redox homeostasis while preserving the adaptive signals that are required for training.

The conceptual framework of this review is presented in Figure 1. The figure illustrates the dual role of exercise-induced ROS/RNS in adaptive signaling and excessive redox stress, and shows how food-based antioxidant strategies may support recovery and redox homeostasis while preserving exercise-induced adaptive signaling.

## 2. Methods

### 2.1. Review Design

This article was written as a narrative review supported by a structured narrative literature search. The review focused on food-based antioxidant nutrition in exercise training, with emphasis on redox signaling, recovery, dietary matrices, and training adaptation. Evidence was collected from exercise physiology, redox biology, sports nutrition, and applied recovery research. This review was not designed as a formal systematic review with rigid eligibility criteria, PRISMA-based screening, protocol registration, risk-of-bias assessment, certainty-of-evidence grading, or meta-analysis. Instead, it aimed to provide an evidence-informed narrative synthesis of how antioxidant-rich foods and dietary patterns may support recovery while preserving redox-sensitive adaptive signaling. Records identified through database and manual searching were first screened for relevance to exercise-induced redox responses, antioxidant nutrition, food-based strategies, recovery, and training adaptation. Potentially relevant articles were then evaluated in full text to determine whether they contributed to the conceptual aims of the review. Final inclusion was based on thematic relevance, methodological quality, human exercise applicability where available, and contribution to mechanistic or practical interpretation. The review was organized around a distinction between high-dose isolated antioxidant supplementation versus food-based antioxidant strategies. Special focus was placed on the mechanism through which redox-active compounds in whole foods, beverages, and dietary patterns may affect oxidative and nitrosative stress, inflammatory regulation, vascular function, gut microbial metabolism, recovery, and cellular signaling pathways related to training adaptation.

### 2.2. Structured Narrative Literature Search

A search of the literature was conducted using PubMed, Web of Science, Scopus, and SPORTDiscus. The primary search focused on literature published between January 2010 and March 2026 to capture contemporary work on exercise-induced redox signaling, antioxidant nutrition, polyphenol-rich foods, nitrate-rich foods, recovery, and training adaptation. However, relevant pre-2010 landmark studies were also considered when they provided foundational mechanistic, methodological, or historical context necessary for interpreting more recent evidence.

Search terms were tailored to each database and were not applied as identical search strings across platforms. This search was multi-faceted, including exercise training concepts, redox biology, antioxidant nutrition, recovery, food matrices, and some food-based sources. Search terms such as “exercise,” “exercise training,” “training adaptation,” “oxidative stress,” “nitrosative stress,” “redox homeostasis,” “reactive oxygen species,” “reactive nitrogen species,” “antioxidants,” “dietary antioxidants,” “polyphenols,” “food-based nutrition,” “whole foods,” “recovery,” “muscle damage,” “inflammation,” “vascular function,” “mitochondrial biogenesis,” “Nrf2,” “AMPK,” “PGC-1α,” “tart cherry,” “berries,” “pomegranate,” “cocoa,” “green tea,” “beetroot,” “dietary nitrate,” “extra virgin olive oil,” and “Mediterranean diet.” Boolean operators were applied as appropriate, but the search was deliberately broad to ensure that the relevant mechanistic, clinical, and applied sports nutrition literature related to the review question was included. Because this article was designed as a narrative review, the search strategy was intended to support conceptual synthesis rather than produce an exhaustive or fully reproducible systematic evidence set.

Database searching was then performed with a manual search of reference lists of pertinent systematic reviews, meta-analyses, consensus statements, position statements, and key original studies. Sources were included when they directly addressed at least one of the following domains: exercise-induced redox responses; antioxidant, polyphenol-rich, or nitrate-rich nutrition; food matrix or bioavailability considerations; recovery outcomes; vascular or inflammatory regulation; gut or oral microbial metabolism; or training adaptation in exercise-related contexts. Supplementary Table S1 summarizes the structure of the search concepts, representative search strings, and how they were organized and used in the narrative synthesis.

### 2.3. Evidence Identification Framework

Evidence was identified and organized according to several interrelated domains, including exercise-induced redox responses, antioxidant nutrition, food-based dietary sources, food matrix and bioavailability considerations, microbial metabolism, training effects, and practical periodization. This framework was used to maintain a focused search while providing sufficient range for narrative synthesis.

Sources were considered relevant, for example, when they contributed to exercise-induced oxidative or nitrosative stress, redox-sensitive signaling pathways involved in recovery and training adaptation, antioxidant supplementation and exercise response, food-based antioxidant techniques, polyphenol-rich foods, nitrate-rich foods, exercise recovery, inflammation, muscle damage, mitochondrial adaptation, vascular function, gut microbial metabolism, as well as exercise-related physiological/performance/athletic nutrition applications.

When practical recommendations were addressed, studies on human exercise and sports nutrition were prioritized. Randomized controlled trials, crossover trials, systematic reviews, meta-analyses, consensus statements, and statements of position were accorded special consideration where they were available. Mechanistic, animal, and cellular research was selected for providing details on biological plausibility or a basis for interpreting human exercise and nutrition responses. Sources were not prioritized when they were unrelated to exercise training or recovery, focused only on disease treatment without clear exercise or nutritional relevance, did not address antioxidant or redox-related mechanisms, or were editorials, opinion pieces, conference abstracts, or non-peer-reviewed materials. English-language peer-reviewed publications were prioritized; however, non-English articles were considered when they appeared directly relevant, had sufficient information available through an English abstract or reliable translation, and contributed important mechanistic or applied evidence.

### 2.4. Evidence Organization and Narrative Synthesis

The evidence was grouped according to the major themes of the review: exercise-induced redox signaling, high-dose antioxidant supplementation, food-based antioxidant strategies, food matrix effects, bioavailability, gut microbial metabolism, recovery outcomes, vascular function, inflammatory regulation, and context-specific practical application. The delivery of antioxidant strategies, including isolated supplements, concentrated extracts, juices, whole foods, beverages, and broader dietary patterns were particularly emphasized. This distinction was central to how we interpreted the evidence.

Findings were synthesized narratively, rather than statistically, because the evidence base varied widely between study populations, exercise modalities, nutritional interventions, dosing strategies, food forms, timing protocols, outcome measures, and mechanistic endpoints. When findings were inconsistent across randomized trials, crossover studies, systematic reviews, meta-analyses, and mechanistic studies, evidence was interpreted according to study design, sample characteristics, training status, intervention form, dose, timing, duration, comparator condition, and outcome specificity. Human exercise studies, systematic reviews, meta-analyses, randomized controlled trials, and crossover trials were prioritized when they directly addressed the relevant food source, exercise context, and outcome. Mechanistic, animal, and cellular studies were used mainly to support biological plausibility and to interpret potential pathways, rather than to override human exercise evidence. The synthesis was approached through three interpretive levels. Mechanistic evidence was first used to describe how exercise-induced reactive oxygen and nitrogen species may contribute to both adaptive and damaging effects. Next, nutritional evidence was employed to differentiate high-dose isolated antioxidant supplementation and food-based antioxidant approaches. Third, applied evidence was incorporated into the generation of context-specific recommendations for athletes, active adults, older adults, and individuals exposed to high recovery demands.

The central interpretation was that food-based antioxidant strategies may support recovery and the return to redox homeostasis without necessarily suppressing adaptive signaling required for training adaptation. Since this article is reported as a narrative review, the results should be read as an evidence-informed conceptual integration, not a quantitative estimate of intervention effects.

## 3. Exercise-Induced Redox Signaling and Training Adaptation

### 3.1. Exercise-Induced ROS and RNS Production

Exercise modifies redox biology through simultaneous increases in energy turnover, oxygen flux, mechanical stress, and inflammatory activity. Although skeletal muscle is the most obvious site of this response, the vascular endothelium, immune cells, liver, adipose tissue, and nervous system also contribute to the systemic redox environment during and after exercise [1,2,5]. Increased mitochondrial respiration supports ATP demand in contracting muscle, but electron leakage from the electron transport chain may also contribute to superoxide formation, particularly during high metabolic flux or altered oxygen and substrate availability [1,2,5].

Other sources of reactive species are also relevant. NADPH oxidases, xanthine oxidase, phospholipase A2-dependent pathways, and uncoupled nitric oxide synthase are other pathways that enhance ROS or RNS production under specific exercise conditions [1,5,11]. Mechanical loading, calcium handling, ischemia–reperfusion-like changes, and inflammatory responses following strenuous or muscle-damaging exercise may also upregulate the redox response, so that it may be amplified. In cases of damaged or stressed tissue, immune-cell recruitment adds another layer because reactive species participate in both defense and repair processes [6,7,8]. Nitric oxide-related pathways also play a role in exercise. Nitric oxide derived from endothelial and neuronal nitric oxide synthases promotes vasodilation, regulates blood flow, glucose uptake, and contractile function. However, peroxynitrite formation may increase nitrosative stress when nitric oxide reacts with superoxide [5,12,13].

The magnitude and physiological meaning of the redox response vary substantially across exercise conditions. Acute high-intensity exercise, prolonged endurance exercise, eccentric contractions, heat stress, hypoxia, and insufficient recovery are more prone to a large redox disturbance than moderate exercise performed within an individual’s adaptive capacity [2,3,4,6]. The response is also altered by training status. Trained individuals generally have more developed endogenous antioxidant defenses and more efficient redox regulation than untrained individuals. Nevertheless, highly trained athletes may experience significant oxidative and inflammatory stress during extended periods of heavy training or during congested competition periods [2,3,4,6,7,8]. For this reason, exercise-induced ROS/RNS production should not be considered a uniform event; it reflects the combined effects of intensity, duration, mode of exercise, environmental stress, nutrition, recovery status, and personal physiological capacity.

### 3.2. ROS/RNS as Adaptive Signaling Molecules

ROS and RNS are commonly related to cellular injury, but this concept is only partially helpful in exercise physiology. Small and transient redox disturbances after exercise do not necessarily indicate failed recovery. Instead, they may form part of the physiological signal linking an acute training bout to subsequent adaptation. Reactive species can play an important role in regulating gene expression, enzyme function, mitochondrial remodeling, vascular function, glucose metabolism, and resolution of inflammation [3,4,5,9]. The identical biochemical family of molecules can thus be distributed on both sides of the training response: beneficial when the signal is moderate and properly resolved and toxic when excessive or unresolved [3,4,9].

A hormesis-based model is therefore more appropriate than a simple damage model. However, the optimal “dose” of redox signaling remains difficult to quantify in humans, and the threshold between adaptive and maladaptive responses may vary according to exercise mode, training status, recovery capacity, nutrition, and environmental stress. A tolerable redox stimulus may support antioxidant defense, mitochondrial function, vascular responses, and metabolic flexibility, whereas excessive or poorly recovered stress may shift the response toward fatigue, inflammation, and impaired function [3,4,10,11]. This dose-dependent interpretation is crucial because it renders endogenously suppressing all the exercise-induced reactive species a dubious target in phases designed to enhance endurance capacity, mitochondrial function, or metabolic health, respectively [14,15,16,17].

The adaptive mechanism of ROS/RNS is also tissue-specific. In skeletal muscle, ROS can aid mitochondrial biogenesis, protein turnover, glucose transport, and endogenous antioxidant protection [3,4,10,11]. NO-related signaling is closely linked to blood flow regulation, mitochondrial respiration, and contractile function [5,12,13]. In vascular, immune, and connective tissues, redox signaling is activated by endothelial adaptation, inflammatory responses, tissue healing, and remodeling [6,7,8] after exercise stress. Therefore, the key issue in sports nutrition is not whether redox signaling matters. Rather, its relationship with health and performance depends on magnitude, timing, recovery status, and training context. The relevant question is whether redox responses occur at an appropriate magnitude and timing under conditions that permit recovery.

This interpretation directly informs antioxidant strategy selection. If reactive species help fuel adaptation, then nutrition must not be organized to eliminate them as completely as possible. A defensible goal is to alleviate the excessive oxidative or nitrosative damage involved while preserving sufficient redox signaling during training induction to develop the relevant adaptation [14,15,16,17]. This is the rationale for differentiating this food-associated antioxidant nutrition from chronic high-dose supplementation with isolated antioxidants.

### 3.3. Nrf2, AMPK, PGC-1α, and Mitochondrial Adaptation

Nrf2, AMPK, and PGC-1α are not presented here as complete explanations for all aspects of training adaptation. Their significance is more specific: they illustrate why antioxidant nutrition cannot be judged solely by whether an intervention lowers oxidative stress markers. However, evidence from cellular, animal, and mechanistic studies should be extrapolated cautiously to athletes because these pathways provide biological plausibility rather than direct proof of performance or adaptation outcomes in human training settings. Exercise-induced redox signals interact with endogenous antioxidant defense, cellular energy sensing, and mitochondrial remodeling, all of which represent contributions to the adaptation that training is expected to elicit [3,4,10,11].

Nrf2 is a helpful illustration of this as it links redox stress with endogenous antioxidant capacity. Oxidative or electrophilic stress induces Nrf2 translocation to the nucleus, where it activates genes involved in glutathione metabolism, heme oxygenase-1, superoxide dismutase, catalase, and related cytoprotective systems [10,11]. The implications for exercise nutrition are straightforward. Training adaptation is not facilitated by just adding exogenous antioxidants from food or supplements. Repeated exercise also strengthens internal redox defense, and the endogenous response may be less desirable to blunt during a training program that targets adaptation [10,11].

AMPK and PGC-1α also warrant similar attention in energy metabolism. AMPK acts on cellular energy stress and is involved in energy restoration, fatty acid oxidation, glucose uptake, and mitochondrial remodeling [3,4,10]. PGC-1α directs transcriptional programs associated with mitochondrial biogenesis, oxidative metabolism, angiogenesis, and fiber-type remodeling [3,4,10]. These pathways are mediated by a number of upstream signals, such as calcium-dependent signaling, p38 mitogen-activated protein kinase [3,4,10,11], AMPK activity [3,4,10,11], and redox-sensitive signaling [3,4,10,11]. Therefore, if antioxidant exposure strongly suppresses the redox component of this network, it may reduce part of the stimulus contributing to metabolic or mitochondrial adaptation, although the magnitude and practical relevance of this effect in athletes remain context-dependent [14,15,16,17].

This does not mean that all antioxidant support is inappropriate. The problem lies in form, dose, timing, and training context. Chronic high-dose isolated antioxidant supplementation may not be well suited to training phases characterized by redox-sensitive signaling that is part of the desired stimulus [14,15,16,17]. In contrast, inadequate dietary antioxidant intake or excessive training stress may increase oxidative damage and impair recovery [1,2,6,7,8]. Food-based antioxidant approaches are more relevant as they may also provide a less pharmacological form of redox modulation. Their potential benefit is not to remove exercise-induced redox activity, but to restore a recoverable redox environment during physiological stress.

### 3.4. When Redox Signaling Becomes Oxidative Damage

Redox signaling is beneficial when the response remains recoverable. When ROS/RNS production is excessive, prolonged, or poorly regulated, the response to exercise can switch to that of oxidative or nitrosative injury. This becomes more likely in the presence of high training stress alongside insufficient energy intake, inadequate sleep, psychological stress, illness, heat exposure, hypoxia, or dense competition schedules [1,2,6,7,8]. In these states, reactive species may outstrip endogenous antioxidant defenses and become involved in lipid peroxidation, protein oxidation, DNA injury, mitochondrial dysfunction, and impaired cellular repair [1,2,5].

In skeletal muscle, unresolved redox disturbance may impair contractile function and delay recovery. Oxidative modification of proteins involved in calcium handling, excitation-contraction coupling, and mitochondrial respiration may reduce force production and increase fatigue [1,2,6]. After strenuous or unfamiliar eccentric exercise, oxidative stress can also interact with inflammation and structural muscle damage, contributing to soreness and temporary reductions in strength or performance [6,7,8]. These responses are not inherently deleterious, as inflammation and redox activity also participate in tissue repair. The concern is persistence, excess, and poor resolution. For athletes, the distinction between adaptive redox signaling and unresolved redox disturbance has practical consequences.

High workloads are required for adaptation, but repeated high-load exposure with insufficient recovery can increase the risk of non-functional overreaching, illness, muscle damage, and performance decline [6,7,8]. Antioxidant-rich foods may be useful in this context, especially when training load, travel, environmental stress, or competition demands increase recovery pressure. The goal is not to erase redox activity. The purpose is to help re-establish a redox environment within the athlete to recover and maintain training signals necessary for adaptation [14,15,16,17].

High-dose isolated antioxidants and food-based strategies should therefore not be evaluated as equivalent interventions. Isolated antioxidants may produce stronger and more immediate antioxidant effects, but this potency may also increase the risk of interfering with redox-sensitive adaptation under certain conditions [14,15,16,17]. Whole foods deliver multiple bioactive compounds within a nutritional matrix and are therefore expected to produce more moderate, diverse, and context-dependent effects [18,19]. Thus, antioxidant nutrition must be appropriate to the phase of training, recovery rate, baseline diet quality, dosage, timing, and personal responsiveness.

In practical terms, adaptive redox signaling is more likely to predominate when exercise stress is moderate, transient, and followed by adequate recovery, energy availability, sleep, and dietary support. Maladaptive oxidative or nitrosative stress is more likely when high-intensity, prolonged, eccentric, or environmentally stressful exercise is repeated with insufficient recovery or inadequate nutrition. Although precise thresholds are difficult to define in humans, the balance appears to depend on the interaction among exercise load, recovery status, nutritional context, environmental stress, and individual adaptive capacity. In sports nutrition, the task is not to silence redox biology, but to recognize when redox stress no longer supports adaptive signaling and instead becomes a recovery problem. This distinction provides the physiological rationale for using food-derived antioxidant nutrition selectively rather than routinely.

## 4. Dietary Antioxidants: Isolated Supplements vs. Food-Based Strategies

### 4.1. The High-Dose Antioxidant Supplementation Controversy

It is easy to understand the appeal of antioxidant supplementation in exercise. Strenuous exercise may lead to enhanced generation of reactive oxygen and nitrogen species and, in some situations, temporarily exceed endogenous antioxidant capacity. In heavy training blocks, repeated competitions, environmental stress, or periods of poor dietary quality, supplemental antioxidants may seem like a direct way to reduce oxidative stress, muscle damage, inflammation, and fatigue [6,7,8,15,17,20]. The challenge is that exercise-induced redox stress is not just a problem of recovery. It may also be part of the adaptive stimulus. Therefore, greater antioxidant exposure does not necessarily translate into better recovery or stronger training adaptation.

The greatest concern is with chronic usage of high-dose isolated antioxidants. Vitamin C and vitamin E have received special interest since increased supplemental doses may reduce redox-sensitive signaling involved in mitochondrial biogenesis, endogenous antioxidant enzyme expression, and metabolic adaptation [14,15,16,17]. Certain studies have indicated that chronic uptake of high-dose antioxidant supplementation may mitigate specific adaptations to endurance or resistance training, but the effects are non-homogeneous between populations, training protocols, supplement regimens, and outcome measures [14,15,16,17]. The evidence does not justify a simple claim that antioxidant supplementation is harmful, but it also does not support the assumption that more antioxidant intake is always better.

A more nuanced interpretation is required because oxidative stress has often been treated as a one-dimensional negative target. Reactive species also contribute to Nrf2, AMPK, PGC-1α, and other pathways, so maximal suppression may remove part of the training signal [3,4,10,11,15]. Oxidative damage also retains relevance, particularly when stress related to the training phase surpasses the capacity to recover. The objective is not the removal of redox activity, but rather to maintain the athlete in a recoverable range. Dose, duration, timing, chemical form, and training stage can substantially influence outcomes. For example, a short-term recovery strategy may be appropriate during congested competition, whereas routine use of the same strategy during an adaptation-driven training block may be less desirable.

A supplement-based model is also not applicable to dietary consumption of antioxidant compounds. Concentrated food extracts and food-derived products occupy an intermediate position between these categories. Concentrated juices, extracts, powders, or standardized polyphenol- or nitrate-rich formulations may retain some food-derived bioactive complexity, but their dose, processing, and bioactive concentration may differ substantially from habitual whole-food intake. A capsule containing either high-dose vitamin C or vitamin E is a narrower intervention. In contrast, foods and meals provide multiple nutrients and bioactive constituents within a dietary matrix [18,19,20,21]. Findings from capsule-based studies should not be transferred to whole foods or dietary patterns. The more useful question is not whether antioxidants are inherently beneficial or detrimental, but whether a given strategy fits the training context, recovery needs, dietary baseline, and adaptation goals [21,22].

**Figure 2:**
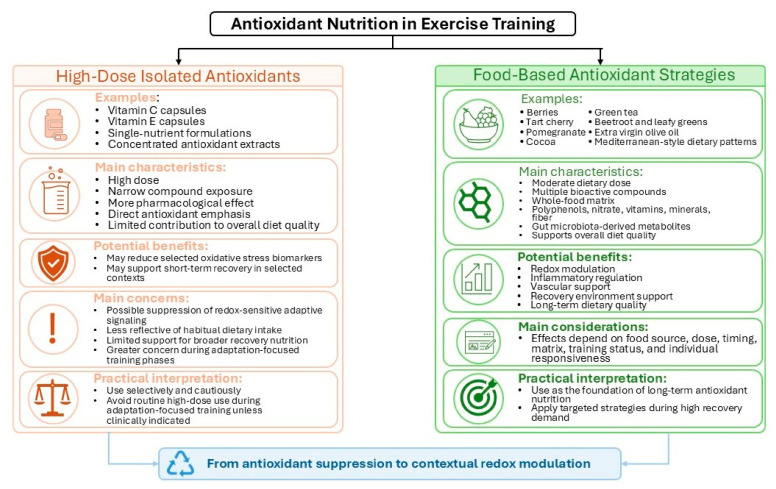
Conceptual shift from high-dose antioxidant supplementation to food-based redox modulation in exercise nutrition. High-dose isolated antioxidant supplementation and food-based antioxidant strategies should not be interpreted as equivalent interventions. Isolated antioxidants, such as high-dose vitamin C or vitamin E, provide a narrow and often pharmacological exposure that may reduce selected oxidative stress biomarkers but may also suppress redox-sensitive adaptive signals under some conditions. In contrast, food-based strategies provide redox-active compounds within complex nutritional matrices, including polyphenols, nitrate, vitamins, minerals, fiber, and other bioactive components. These foods may influence recovery, vascular function, inflammatory regulation, gut microbial metabolism, and overall diet quality through moderate and multi-targeted mechanisms. This distinction supports a shift from indiscriminate antioxidant suppression toward contextual redox modulation. The orange panel represents high-dose isolated antioxidant supplementation, whereas the green panel represents food-based antioxidant strategies. Arrows indicate the conceptual shift from antioxidant suppression toward contextual redox modulation.

Figure 2 abstracts the conceptual separation between high-dose isolated antioxidant supplementation and matrix-based dietary approaches. This figure illustrates why these interventions should not be treated as equivalent. Table 1 further summarizes the practical and mechanistic differences between high-dose isolated antioxidant supplementation and food-based antioxidant strategies, with particular attention to their implications for training adaptation.

### 4.2. Whole Foods as Redox-Modulating Nutritional Matrices

One core limitation of applying antioxidant-supplement evidence to foods is that whole foods are not simply low-dose versions of isolated nutrients. Fruits, vegetables, nuts, seeds, cocoa, tea, extra virgin olive oil, whole grains, and other plant-based foods contain polyphenols, carotenoids, vitamin C, vitamin E, minerals, fiber, nitrate, organic acids, and other bioactive constituents in complex nutritional matrices [18,19,20]. These matrices shape the release, absorption, metabolism, and pairing of compounds with other dietary components. Food structure can influence biological responses rather than simply delivering antioxidant molecules.

The same logic holds when interpreting certain foods. Berries contain anthocyanins, vitamin C, fiber, and other phenolic compounds. Tart cherry provides anthocyanins and related polyphenols that researchers have studied in terms of inflammation and muscle soreness. Cocoa flavanols may influence endothelial function and vascular responses. Beetroot contains nitrate, betalains, and other compounds associated with nitric oxide availability and redox balance [20,23,24,25]. The strength of evidence is not equivalent across these food sources. Evidence appears relatively stronger for selected targeted outcomes, such as tart cherry in relation to muscle soreness and functional recovery and beetroot or nitrate-rich vegetables in relation to nitric oxide-related vascular function and exercise efficiency, whereas findings for berries, cocoa, green tea, pomegranate, extra virgin olive oil, and Mediterranean-style dietary patterns remain more heterogeneous or context-dependent. These cases demonstrate that antioxidant capacity alone is a weak criterion for assessing dietary interventions. These effects on recovery and performance could include antioxidant defense, inflammatory regulation, vascular function, mitochondrial signaling, and gut microbial metabolism simultaneously [18,19,20,26,27].

This matrix-based perspective also changes how whole foods should be compared with high-dose supplementation. Whole foods normally offer the antioxidant compound in lower, more distributed, and more physiologically integrated forms than capsules or isolated extracts. Their effects are unlikely to recapitulate the potent pharmacological suppression suggested for some high-dose products [15,18,19,20]. In parallel with this, they add to the overall quality of the diet, energy availability, carbohydrate intake, protein distribution, micronutrient adequacy, and gastrointestinal health, which directly affect recovery and adaptation [21,22]. Therefore, food-based strategies should be evaluated in relation to recovery, adaptation, diet quality, and training context, not only by changes in oxidative stress markers.

This argument does not exclude the role of supplementation in sports nutrition. Supplements, sports foods, and concentrated products may be helpful, particularly in situations where travel, gastrointestinal tolerance, competition schedules, and/or availability of food do not permit access to whole foods or specific nutrient requirements make whole-food intake difficult. The point is narrower: for long-term support of training, antioxidant-rich foods might provide a more integrative option as they provide redox-active compounds while also providing the dietary basis for recovery, immune function, and adaptation [21,22]. In this review, these foods are considered as part of a more total nutritional plan, instead of in isolation as measures to suppress oxidative stress.

### 4.3. Polyphenols Are Not Merely Antioxidants

Describing polyphenols simply as antioxidants is useful but incomplete in exercise nutrition. Many polyphenols have shown that they can scavenge free radicals under laboratory conditions, but concentrations within human tissues after intake are typically insufficient for understanding their effects through direct radical scavenging alone [18,19]. Their relevance to exercise may depend more on cellular signaling, enzyme regulation, vascular function, inflammatory modulation, mitochondrial regulation, and interactions with gut microbial metabolism [18,19,20].

This distinction is important because ROS and RNS induced by exercise are not undesired byproducts. Moderate redox signals are involved in adaptation during training, and therefore, a helpful nutrition approach should not be described primarily by its intensity of reduction in oxidative stress. Polyphenol-dense foods are more readily regarded as potential modulating agents for the exercise-related response. Anthocyanin-containing foods may affect the level of inflammatory indicators and muscle soreness after exercise. Cocoa flavanols can influence endothelial function and blood flow. Green tea catechins may influence oxidative stress, fat oxidation, and inflammatory regulation. Pomegranate polyphenols might influence recovery and vascular function. The effects of these agents are unlikely to derive solely from one antioxidant mechanism [20,23,24].

Several pathways make this interpretation biologically plausible. However, the human mechanistic evidence remains incomplete. Many proposed pathways are supported by cellular, animal, biomarker-based, or indirect mechanistic evidence, whereas direct confirmation in human exercise settings remains limited and may vary according to dose, food form, training status, and outcome domain. Foods rich in polyphenols and their metabolites may affect Nrf2-related antioxidant defense and NF-κB-related inflammatory signaling, in addition to NO bioavailability and mitochondrial regulatory pathways [18,19,20,26,27]. The response, however, is not consistent for foods or for individuals. The effect of a given food on an individual is influenced by source, dose, timing, duration of intake, training status, gut microbial metabolism, and baseline diet [20,23,24]. This variability is not simply a methodological limitation; it is central to interpreting the current evidence on polyphenol-containing foods in exercise settings. Variation in food source, dose, timing, duration of intervention, exercise model, training condition, the quality of baseline diet, and production of gut-derived metabolites may be the reason that some studies report large, significant recovery or performance improvements, but others report small, null, and variable effects.

Polyphenol-containing foods should, therefore, not be described as uniform ergogenic aids. Their practical value is most likely only when exercise leads to significant muscle damage, tissue inflammation, oxidative stress, or vascular strain, and it is potentially less evident in moderate loads of training or high dietary quality. In practice, they are most relevant during long training sessions, prolonged periods of high training intensity, short recovery intervals between demanding sessions, or periods of suboptimal diet quality.

### 4.4. Food Matrix, Bioavailability, and Gut Microbial Metabolism

The biological effects of antioxidant-rich foods are not determined solely by their antioxidant content. Bioavailability and metabolism are just as important. Most polyphenols are poorly absorbed as their original substrate and undergo substantial conversion in the small intestine, liver, and colon after ingestion. The gut microbiota are crucial in this process because they metabolize the larger polyphenolic compounds into smaller metabolites, which may serve as bioavailable or bioactive constituents [18,19,26,27]. These metabolites can then act on inflammation, endothelial status, mitochondrial regulation, and redox-sensitive signaling.

This microbial step may also explain some inter-individual variation in responses to polyphenol-rich foods. For example, two athletes consuming the same food may develop different metabolite profiles because of differences in gut microbiota composition, habitual diet, training status, age, health status, or recent antibiotic exposure [18,19,26,27]. In sports nutrition, intake alone cannot quantify exposure. Instead of assuming that all participants’ biological responses are the same for the same food, future research should take into account metabolite production, gut microbial ecology, and responder and non-responder profiles. This issue is likely to become a major direction for future research because average group responses may obscure meaningful individual differences. Future studies should therefore consider baseline diet, gut microbial profiles, oral microbiota, metabolite production, training status, sex, age, and intervention form when evaluating responsiveness to food-based antioxidant strategies. It is this inter-individual variability that might partly explain why polyphenol-rich foods produce distinct recovery or vascular effects in certain trials, but relatively modest or no clinical effects in others.

For example, cocoa flavanol responses may vary according to processing methods, flavanol preservation, and product composition, including sugar and fat content [23]. Nitrate-rich vegetable responses are influenced by nitrate dose, oral nitrate-reducing bacteria, product form, and antibacterial mouthwash use [12,13,25]. Extra virgin olive oil may exert effects through phenolic molecules, monounsaturated fatty acids, and broader dietary-pattern interactions [26,27]. These examples point to the fact that whole-food interventions should not be considered supplements in isolation.

For applied sports nutrition, antioxidant-rich foods are more appropriately recognized as targeted foods or dietary-pattern agents than as simple carriers of antioxidant molecules. The same food may be used differently during an adaptation-focused training block, before competition, after muscle-damaging exercise, or as part of a long-term dietary pattern. When recovery demands are greater, certain choices may be more relevant, whereas others may primarily support baseline diet quality and redox homeostasis [20,21,23,24,25]. The relevant issue is not whether a food has antioxidant capacity in isolation, but whether it helps establish a physiological environment that supports recovery and adaptation.

In contrast, whole foods, polyphenols, and dietary matrices provide an alternative to supplementation-centered approaches. Their effects are likely to be moderate, multi-targeted, and context-dependent [18,19,20]. This perspective supports the examination of specific dietary sources, including berries, tart cherry, cocoa, pomegranate, green tea, beetroot, extra virgin olive oil, and Mediterranean-style dietary patterns, in relation to exercise recovery and training adaptation.

## 5. Food-Based Strategies for Exercise Recovery

Food-based antioxidant interventions appear most appropriate when exercise recovery presents a clearly defined problem, rather than as a routine daily practice. Following difficult physical training or competition, recovery may be constrained by muscle damage, inflammatory activity, vascular strain, substrate depletion, neuromuscular fatigue, and incomplete restoration of function [6,7,8,20,21]. Antioxidant-rich foods should not be considered natural substitutes for high-dose supplementation in this context. Their importance rests upon the food matrix, dose, timing, baseline diet quality, recovery requirement, and physiological target of the intervention [18,19,20].

The strongest practical rationale emerges when soreness, inflammation, or short-term functional decline threatens the quality of the training plan. Tart cherry is most extensively studied in this context, especially after extreme exercise like endurance work, resistance work, repeated sprint exercises, and muscle-damaging exercise [28,29,30,31,32]. A related but more heterogeneous evidence base is presented in the case of berries (blueberries and blackcurrants), which, under particular conditions, have been connected to the recovery of muscle function, oxidative stress, gut-derived phenolic metabolites, vascular function, and the performance of the exercise [20,27,33,34,35,36,37]. Such inconsistency may be ascribed to variations in the muscle damage caused by exercise; product form; polyphenol dosage; timing of intake; duration of the intervention; training status; and recovery outcomes selected for measurement. Thus, these foods are best understood as targeted recovery options for high-recovery-demand conditions, not as generic ergogenic aids.

Other food-based approaches are more closely related to vascular control, nitric oxide availability, metabolic regulation, or long-term diet quality. Cocoa flavanols, pomegranate polyphenols, green tea catechins, beetroot, and nitrate-rich vegetables are studied for their potential for use in the management of endothelial function, blood flow, oxidative stress, inflammation, fat oxidation, exercise efficiency, or repeated high-intensity exercise capacity [23,24,25,26,38,39,40,41,42,43,44,45,46,47,48,49,50]. However, the evidence remains mixed, and this variability may depend on product formulation, bioactive compound profile, dose, timing, training status, oral or gut microbiota, and exercise model. This is particularly relevant for beetroot and nitrate-rich vegetables because their effects cannot be attributed solely to nitrate: betalains, polyphenols, minerals, product processing, and oral nitrate-reducing bacteria (and by extension, baseline training status) can modify the physiological response. Hence, these foods should be matched to the physiological problem being targeted, not placed together around a common antioxidant label.

Dietary patterns provide a wider platform for recovery than any specific food. Extra virgin olive oil and Mediterranean diet practices may have a role in redox function, inflammatory control, vascular function, gut condition, and overall nutrition [51,52,53,54]. For an athlete, however, these patterns must still be adjusted to sport-specific energy, carbohydrate, protein, hydration, and micronutrient needs [21,51,55,56]. This is important because recovery capacity is built on daily nutrition rather than the addition of any single recovery-focused food.

The practical implication is context-dependent. Food-based antioxidant strategies are most helpful during heavy training blocks, congested competition, muscle-damaging exercise, environmental stress, travel, and periods of lower dietary variety [20,21,28,51]. These strategies should be considered as potential adjuncts to, rather than replacements for, the cornerstones of sports nutrition, including adequate energy availability, carbohydrate intake, protein distribution, hydration, and sleep [21,22,55,56]. Table 2 displays a summary of the major food-based antioxidant sources, potential mechanisms to account for them, exercise-related outcomes, and evidence considerations.

## 6. Balancing Recovery and Adaptation

The practical question is whether antioxidant nutrition is appropriate for the purpose of the current training phase. Exercise-related reactive oxygen and nitrogen species induce fatigue, soreness, inflammation, and slower recovery if generated in higher amounts, but can be associated with signaling pathways promoting mitochondrial biogenesis, endogenous antioxidant defense, vascular adaptation, and skeletal muscle remodeling [3,4,9,10]. That would establish a small physiological margin within which recovery support and adaptive stimulation should be balanced. A short-term recovery strategy is not necessarily optimal for long-term adaptation, particularly if it strongly inhibits redox-sensitive signaling [14,15,16,17]. Antioxidant nutrition should, therefore, be evaluated in terms of training phase, recovery requirement, food form, dose, timing, and individual responsiveness.

### 6.1. Training Phase Matters

Antioxidant nutrition should vary across the training year. The principal objective of base training or adaptation-focused periods is to promote major physiological remodeling, which includes improvements in mitochondrial activities, aerobic capacity, metabolic flexibility, muscle strength, and tissue tolerance. In that context, one of the goals of training stimulus is exercise-induced redox signaling from exercise [3,4,9,10]. Excessive intake of isolated antioxidant supplements may be inappropriate during phases designed to promote specific adaptive responses [14,15,16,17]. A rational approach is to put an emphasis on the quality of the pattern of meals, energy availability, food intake involving carbohydrates and protein, and a high variety of fruits, vegetables, whole grains, legumes, nuts, seeds, and foods rich in nutrients [21,22,51,55,56].

During intense training blocks, the emphasis may shift toward recovery support. Sharp increases in the training load or minimal recovery period may exacerbate muscle soreness, inflammation, sleep disturbance, immune activity, and fatigue accumulation [6,7,8,57,58]. Within this framework, food-based antioxidant interventions might provide athletes with a means of preserving training quality, without attempting to eliminate the adaptive signal generated by training. Tart cherry, pomegranate, berries, and other polyphenol-rich foods may be beneficial in the moment when muscle damage and soreness would reasonably threaten the next session [20,28,29,30,31,32,40]. The goal is not to prevent training stress, but to improve tolerance to repeated loads and maintain training continuity. Competition periods require a different logic. In tournaments or events involving repeated bouts, the immediate goal is often rapid restoration of performance capacity rather than maximizing long-term adaptation. Within this context, short-term selective use of antioxidant-rich foods may be reasonable. Tart cherry, pomegranate, nitrate-rich vegetables, and other recovery-promoting foods may be used before or after competition to reduce soreness, support vascular function, and facilitate repeated performance [28,32,40,48,49]. In addition, concentrated antioxidant products or selected antioxidant supplements may be considered during congested competition periods when competitive events are scheduled in close proximity and rapid between-bout recovery is the main priority. Such use should be targeted and short-term, rather than routine during adaptation-focused training. Because acute readiness is the primary goal in these settings, concern about attenuating long-term training signals is less prominent than during an adaptation-focused training block.

Recovery and transition phases have specific nutritional requirements. Antioxidant-rich dietary habits may help restore physical, immune, and inflammatory balance after a competition season, injury, illness, or a period of high cumulative load [21,22,25,51]. These approaches must still be combined with adequate energy intake, protein distribution, sleep, and load control [21,22,58,59]. Antioxidant-rich foods cannot compensate for chronic under-recovery or poor training design, but they may become more useful when incorporated into an overall recovery or rehabilitation plan.

### 6.2. Recovery Support Versus Adaptive Signaling Preservation

Recovery and adaptation are often discussed together, but they are not the same. Recovery is about returning to function after exercise, with activities including glycogen resynthesis, fluid replacement, muscle repair, nervous system recovery, immune regulation, and the resolution of inflammation [6,7,8,21,57,58,60]. Adaptation is the long-term remodeling that permits the body to endure and act more effectively under future stressful situations. For this remodeling to occur, some degree of physiological stress is required. When every stress signal is attenuated too rapidly, some information is lost in the training stimulus [3,4,14,15,16].

This distinction is important when interpreting antioxidant interventions. Some food-based strategies may potentially support both recovery and adaptation when they improve training continuity, nutrient availability, vascular function, and inflammatory resolution without strongly suppressing redox-sensitive signaling. A diminished oxidative stress biomarker after antioxidant intake is not necessarily evidence of better training adaptation. It may only indicate that the acute redox disturbance was reduced. That effect can be useful following any damaging competition or repeated exposure to a match because the goal is to recover performance capacity. When training a block intended to promote mitochondrial biogenesis or to remodel metabolism, the same suppression level would probably be less desirable [14,15,16,17]. Even reduced muscle soreness is to be taken with caution. This may aid the athlete in completing the next session, but it does not necessarily signify superior long-term adaptation [6,7,8,28].

Food-based strategies may be useful because they act within a broader recovery context rather than through a single strong biochemical effect. Whole foods are rich in redox-active compounds, carbohydrates, amino acids, minerals, fluids, fiber, and other bioactive constituents [18,19,20,21]. Together, these modulators would be able to modulate inflammation, promote vascular function, and restore nutrient availability. Even so, food-based approaches must be viewed not as biologically neutral. Their effects are mediated by the food form, dose, timing, training phase, and recovery status, and hence could differ from the narrower pharmacological effects observed from high-dose isolated supplements [18,19,20,21,22].

Therefore, the more useful question is not whether antioxidants should be added, but whether the chosen strategy matches the physiological aim of the training period. The goal is to help the body clear excess inflammation, recover from harmful stress, and to keep a balance of redox without categorizing exercise-induced redox activity as harmful [3,4,15,20]. In practice, a food-based approach is justified only when it is appropriate to the athlete’s current training objective and recovery state.

### 6.3. Dose, Timing, and Duration

The impact of an antioxidant strategy is related to the size of the exposure. Most of the concern about impaired training adaptation results from trials with high doses of isolated antioxidants, especially vitamin C and vitamin E [14,15,16,17]. Those doses should not be viewed as the amounts typically derived from foods. Fruits, vegetables, tea, cocoa, and Mediterranean-style dietary patterns tend to offer lower, diverse, and more spread-out exposure to redox-active compounds [18,19,20]. Results from high-dose supplement studies should be used sensitively with the interpretation of whole-food or dietary-pattern approaches.

Timing further alters the interpretation. Antioxidant intake may have more direct interaction with the acute redox response at the time of intake (as at exercise) than in the consumption of ordinary meals. This might be acceptable, or even helpful, after damaging exercise or repeated competition, when recovery from damaging or repeated exercise is the primary goal. In an adaptation-focused session, however, strong acute antioxidant exposure close to exercise may be less desirable if it attenuates signals involved in mitochondrial or metabolic remodeling [14,15,16,17]. The intake of antioxidant-rich foods across meals is different. In fact, it will help with diet quality and redox resilience rather than focus on one acute exercise signal [21,51].

Duration of use also matters. A quick tart cherry or pomegranate tactic around a race, tournament, or muscle-damaging block has another reason than long-term daily use of concentrated antioxidant supplements [28,29,30,31,32,40]. Short-term usage is warranted when a significant recovery issue is identified. Extended long-term high-dose supplementation during an adaptation-oriented workout may be more difficult to justify, unless there is a targeted clinical or nutritional rationale [14,15,16,17].

The form of intake also needs to be specified. Food, juice, concentrate, extract, capsule, and isolates do not provide equivalent exposure. They differ in quantity, matrix, bioavailability, co-ingested nutrients, and metabolic fate [18,19,20,26,27]. Even in foodborne products, processing will cause changes in polyphenol content, sugar level, fiber content, and physiological effect [23,38,39]. Describing a product as “antioxidant” is therefore not specific enough for sports nutrition planning.

A periodized model is proposed here as a conceptual, evidence-informed approach rather than an evidence-graded guideline. During adaptation-focused training, the priority should be a broad and diverse diet without routine high-dose antioxidant supplementation. During intense training or competition, selected antioxidant-rich foods may be added when recovery demand is high. This approach places antioxidant nutrition within the same logic as training and nutrition periodization: strategies may be increased, reduced, or withheld according to the purpose of each phase, rather than follow a fixed rule that more antioxidant exposure is always better [21,55,56]. For practical application, an endurance athlete in an adaptation-focused training block might maintain a high-quality habitual diet while avoiding routine high-dose antioxidant supplementation, whereas the same athlete during a heavy training week or multi-day competition may increase tart cherry, berries, pomegranate, or nitrate-rich vegetables to support recovery. In team sports, coaches may prioritize familiar portable foods and selected concentrated products during congested match schedules or travel when normal food access, gastrointestinal comfort, and between-match recovery are limiting factors. These examples should be interpreted as context-specific applications rather than fixed prescriptions.

### 6.4. Athlete Type, Training Status, and Individual Responsiveness

Responses to antioxidant nutrition have wide variation between people and types of sports. Endurance athletes, resistance-trained athletes, team-sport athletes, recreationally active adults, and older adults do not necessarily require the same strategy. Endurance training adaptations depend strongly on mitochondrial remodeling, oxidative metabolism, and vascular function. Chronic high-dose antioxidant supplementation may therefore be more concerning during periods designed to promote aerobic adaptation [3,4,14,15,16,17]. Food-based interventions, such as nitrate-rich vegetables, berries, cocoa, and Mediterranean-style dietary patterns, may also support vascular function, recovery, and long-term health when implemented in an appropriate context and form [23,25,28,33,48,49,50,51].

Resistance training and high-intensity exercise present distinct recovery demands. After an unfamiliar eccentric load, high-volume resistance exercise, or repeated sprint activities, muscle injury, soreness, neuromuscular fatigue, and inflammation are common [6,7,8]. Polyphenol-rich foods, including tart cherry, berries, and pomegranate, may be useful in these conditions when soreness or reduced force production compromises subsequent training quality [28,29,30,31,32,33,34,35,36,37,40]. Inflammation and cellular change are also involved in muscle remodeling, and rehabilitation should be thoughtfully applied as part of the recovery training in periods where hypertrophy or strength adaptation is the objective.

Team-sport athletes often face congested schedules, travel, irregular sleep, repeated exertion, fatigue, and limited recovery time [57,58,61,62,63]. Pragmatic, food-based interventions may be particularly relevant for these athletes because recovery between repeated training sessions and match play is often a practical constraint. After training or competition, athletes need balanced recovery nutrition that supports carbohydrate restoration, fluid and electrolyte replacement, protein intake, gastrointestinal comfort, and the use of familiar foods during travel or tournament preparation [21,57,58,60,61,62,63]. In a broader recovery context, tart cherry, nitrate-rich vegetables, fruit-based recovery meals, and high-quality mixed dietary patterns may contribute to recovery, hydration, vascular function, and immune resilience [21,28,32,48,49,57,58,60,61,62,63]. When a competitive team faces a tight schedule with limited time between successive matches, concentrated antioxidant products or selected antioxidant supplements may also be considered for short-term between-bout recovery. This use should be targeted and situation-specific, rather than a default strategy during adaptation-focused training. Although long-term adaptation remains important, recovery between repeated bouts may become the immediate priority.

Another key population is older adults. Older age is frequently related to higher oxidative stress, chronic, low-grade inflammation, reduced mitochondrial function, anabolic resistance, vascular dysfunction, and prolonged recovery [3,4,59,64,65,66]. Food-based antioxidant strategies may be relevant for older adults when combined with sufficient protein intake and resistance or multicomponent exercise training. Polyphenol-rich foods, Mediterranean-style diet models, and nitrate-rich vegetables could support vascular, metabolic, and inflammatory health [25,51,59,64,65,66]. Older individuals still need enough energy and protein to achieve muscle remodeling and to counter anabolic resistance. Antioxidant-rich foods should complement nutrition programs that directly support muscle protein synthesis and functional adaptation [21,59,64,65].

This individual sensitivity continues to be a major limitation within the current evidence base. Responses to polyphenol-rich foods, nitrate-rich foods, and other redox-active dietary strategies may vary according to baseline diet, gut microbiota, oral microbiota, training status, sex, age, genetics, and health status [18,19,26,27,67]. Responses to nitrate-rich foods may depend partly on oral nitrate-reducing bacteria, whereas responses to polyphenol-rich foods may depend strongly on gut microbial metabolism [12,13,25,26,27,67]. Separate dietary strategies that support gut microbial ecology may also influence the effectiveness of antioxidant-rich foods. These may include probiotic-containing fermented foods, such as yogurt, and prebiotic fiber-rich foods, such as lentils, chickpeas, beans, and other pulses. Thus, a strategy that appears beneficial for one athlete may be less effective for another, and future research should focus more on responder profiles rather than average group effects.

Food-based antioxidant strategies need to be individualized in practice. Athletes with high fruit and vegetable consumption may respond differently from those with minimal baseline antioxidant intake. Recreationally active individuals may also require different strategies from highly trained athletes. Recovery and adaptation requirements may further differ according to sex, age, training status, energy availability, and health context [21,22,59,64,65,66,67,68,69,70]. For example, healthy female athletes may not require greater antioxidant exposure by default, and in some contexts may require less aggressive antioxidant targeting if recovery is already adequate. However, current evidence is not sufficient to recommend a universal lower dose for females; decisions should consider training phase, recovery demand, hormonal status, menstrual function, energy availability, and iron status [68,69,70]. Accordingly, sex-specific responses should be considered earlier in practical decision-making, particularly when recovery demand, hormonal status, menstrual function, energy availability, iron status, and training phase may interact with redox balance and antioxidant needs. Therefore, food-based antioxidant strategies should be periodized and individualized rather than applied as a universal recommendation.

**Figure 3:**
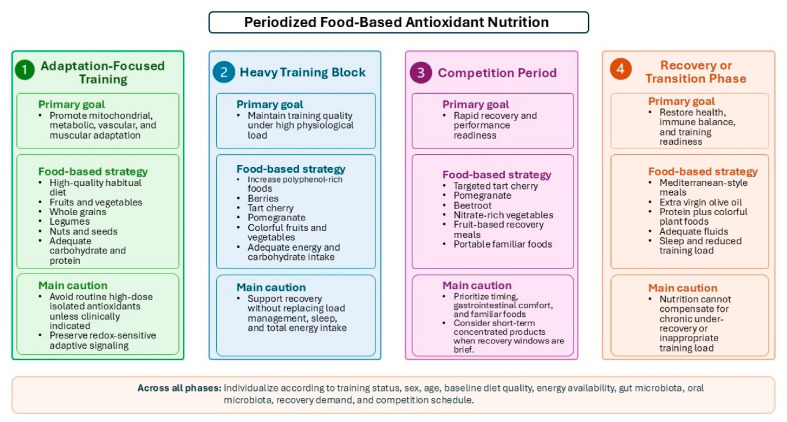
Periodized food-based antioxidant nutrition across training and competition phases. Food-based antioxidant strategies should be applied according to training context rather than used as a fixed daily intervention. During adaptation-focused training phases, the priority is to maintain a high-quality habitual diet while avoiding routine high-dose isolated antioxidant supplementation that may suppress redox-sensitive adaptive signaling. During heavy training blocks, polyphenol-rich foods such as berries, tart cherry, pomegranate, and colorful fruits and vegetables may support recovery and training continuity. During competition periods, targeted use of recovery-supporting foods, including tart cherry, pomegranate, beetroot, nitrate-rich vegetables, and fruit-based recovery meals, may help maintain performance readiness. During recovery or transition phases, Mediterranean-style meals, extra virgin olive oil, adequate protein, colorful plant foods, hydration, sleep, and appropriate load reduction may help restore general health and training readiness. Across all phases, strategies should be individualized according to training status, sex, age, baseline diet quality, energy availability, gut microbiota, oral microbiota, competition schedule, and recovery demand. During congested competition periods, short-term use of concentrated antioxidant products or selected supplements may be considered when successive events are scheduled closely and rapid between-bout recovery is the immediate priority; however, this should not be interpreted as support for routine high-dose antioxidant supplementation during adaptation-focused training. This figure should be interpreted as a conceptual, evidence-informed framework intended to support practical decision-making rather than as a formal evidence-based or evidence-graded guideline. The numbers indicate sequential training or competition phases, and the colors distinguish the four contextual phases: green for adaptation-focused training, blue for heavy training blocks, purple for competition periods, and orange for recovery or transition phases.

Recovery versus adaptation requires a shift in perspective. Antioxidant nutrition should not be employed as a single daily treatment to decrease oxidative stress. It should match the training phase, recovery demands, competition requirements, and individual characteristics. The framework of food-based antioxidant nutrition is particularly relevant here because it promotes dietary quality and redox homeostasis and avoids the excessive supplement-induced suppressive mechanism of adaptive signaling. On this basis, Figure 3 proposes a periodized model for applying food-based antioxidant strategies across adaptation-focused training, heavy training blocks, competition periods, and recovery or transition phases.

## 7. Practical Recommendations

Practical decision-making may begin by identifying the primary nutritional and physiological challenges in a specific training environment. Because exercise-induced redox signaling is part of the target stimulus during adaptation-focused phases, aggressive antioxidant targeting may not be necessary in many adaptation-focused contexts [3,4,9,10,14,15,16,17]. When there is a great deal of congested competition, muscle-damaging exercise, travel, heat exposure, or insufficient dietary variety, the priority might be to restore readiness and to minimize unresolved stress [6,7,8,20,28,57,58,59,60,61,62,63,71,72,73,74,75]. Different situations may require different nutritional decisions. The starting point may be the overall diet rather than a rigid rule. Whole foods, meals, and dietary patterns provide redox-active compounds within nutritional matrices that also support energy intake, carbohydrate availability, protein distribution, hydration, micronutrient adequacy, and overall diet quality. In practical sport contexts, juices, concentrates, sports foods, and selected supplements may still be warranted when travel, gastrointestinal tolerance, competition schedules, or specific nutrient requirements make habitual eating difficult [21,22]. The question is not simply whether a strategy is food-based or supplemental, but whether it matches the athlete’s current recovery demands and adaptation objectives. The practical rationale for antioxidant-rich foods appears strongest under conditions of high recovery pressure. Tart cherry, berries, pomegranate, nitrate-rich vegetables, fruit-based recovery meals, Mediterranean-style dietary patterns may be appropriate in periods of heavy training blocks, repeated competitions, muscle-damaging exercise, environmental stress, and when dietary diversity is limited [20,23,24,25,28,29,30,31,32,33,34,35,36,37,38,39,40,41,42,43,44,45,46,47,48,49,50,51,52,53,54,57,58,59,60,61,62,63,71,72,73,74,75]. In low-stress or adaptation-focused phases, the focus may need to return to habitual diet quality, sufficient energy availability, and sport-specific nutrient timing, rather than additional antioxidant targeting [21,22,55,56]. Accordingly, that food-fueled approach may be suitable for one training scenario but inappropriate for another. Table 3 summarizes how food-based antioxidant strategies and selected concentrated products may be tailored to exercise context, key physiological challenge, proposed mechanism, practical caution, and representative evidence. However, several recommendations in this decision guide rely on indirect evidence, mechanistic reasoning, or extrapolation from related populations rather than intervention trials conducted specifically in each listed population or context; therefore, the table should be interpreted as a conceptual, evidence-informed decision guide rather than as a population-specific intervention guideline. Strategies that may be useful during one phase may not be needed during another. In practice, the goal may be to support recovery during periods of high physiological stress, while avoiding routine inhibition of the redox-sensitive signals involved in training adaptation.

## 8. Limitations and Future Directions

This review should be interpreted with several limitations in mind. It was designed as a narrative review supported by a structured narrative literature search, not as a systematic review or meta-analysis. Although the search strategy was structured, selective inclusion of evidence cannot be fully excluded because no protocol-based screening, formal study selection flow, or risk-of-bias assessment was performed. For that reason, the article does not provide pooled effect estimates, certainty-of-evidence grading, or formal comparative judgments of intervention efficacy. The synthesis is best read as an evidence-informed conceptual integration rather than as a quantitative summary of treatment effects. The literature itself also limits the strength of practical conclusions. Studies differ in exercise modality, training status, food source, product form, dose, timing, duration of intake, comparator condition, and outcome measures. These differences make it difficult to translate the evidence into universal recommendations for food-based antioxidant strategies. In addition, because English-language peer-reviewed publications were prioritized, language bias may have influenced the evidence base considered in this narrative synthesis, although directly relevant non-English studies were considered when sufficient information was available through an English abstract or reliable translation. Publication bias may also have influenced the available evidence, because positive or statistically significant findings may be more likely to be published than null, inconsistent, or practically modest effects.

A second limitation is that much of the available evidence remains focused on short-term recovery outcomes. Soreness, inflammatory markers, oxidative stress biomarkers, and acute performance recovery are useful endpoints, but they do not fully answer the question of how antioxidant-rich foods influence training adaptation over time. Fewer studies have followed athletes across training phases or competitive seasons. As a result, the field still lacks clear evidence on whether repeated use of food-based strategies alters mitochondrial adaptation, strength development, endurance capacity, immune resilience, or performance across a full training cycle. Individual responsiveness is another unresolved issue. Baseline diet quality, training status, sex, age, low energy availability, gut microbiota, oral microbiota, and habitual polyphenol or nitrate intake may all change the response to the same intervention.

The next stage of research needs to move away from the narrow question of whether antioxidant intake reduces oxidative stress. A more useful question is whether a specific food-based strategy improves the balance between recovery and adaptation under a clearly defined training condition. Antioxidant-rich foods can influence recovery, inflammatory regulation, vascular function, and redox homeostasis, but the direction and size of these effects are likely to depend on dose, timing, food form, training phase, baseline diet, and individual responsiveness [18,19,20,28,48,49,50,51]. Trials should therefore be designed to identify when a strategy is useful, for whom it is most effective, and how it can be integrated into periodized sports nutrition plans [21,22,55,56]. Such studies should also examine whether dose- and timing-specific responses differ according to training phase, recovery demand, food matrix, intervention duration, and outcome domain.

Dose and timing require more precise study. Existing trials use different doses, durations, and delivery forms of polyphenol-rich foods, tart cherry, pomegranate, cocoa, green tea, beetroot, and other antioxidant-rich products [20,23,24,25,28,38,40,48,49,50]. This variation makes it difficult to compare findings or convert them into practical recommendations. Future work should distinguish acute use, short-term recovery blocks, and habitual intake across a training cycle. It should also separate strategies used before exercise, immediately after exercise, during heavy training, and during competition. This distinction matters because antioxidant intake close to the exercise stimulus may interact with acute redox signaling differently from antioxidant-rich foods consumed as part of daily meals [14,15,16,17].

Food matrices and whole-diet approaches also require more robust evidence. A great deal of research continues to investigate isolated compounds, concentrated extracts, or single products. Such designs can help shed light on mechanisms, but they do not completely align with how athletes eat. Whole foods are rich in interacting nutrients and bioactive components like polyphenols, nitrate, vitamins, minerals, fiber, fatty acids, and organic acids [18,19,20,21]. These molecules may have effects on digestion, absorption, gut microbial metabolism, vascular function, and inflammatory signaling, unlike those of discrete supplementation [18,19,26,27]. Robust whole-diet trials will be required to determine if dietary patterns high in fruits, vegetables, legumes, nuts, seeds, tea, cocoa, extra virgin olive oil, and nitrate-rich vegetables can support redox balance and recovery across an entire training cycle [21,51].

Sex-specific evidence continues to be scarce. Exercise nutrition research has often focused on male participants or has not been adequately powered to test sex-specific responses. This limits interpretation because hormonal status, menstrual cycle phase, contraceptive use, iron status, substrate metabolism, inflammation, vascular function, and recovery patterns may influence redox responses and antioxidant requirements [68,69]. Female athletes may also have low energy availability, menstrual dysfunction, and an increased risk of iron deficiency that can interact with oxidative stress and recovery [70]. Future studies should draw on sufficiently well-powered female cohorts, report menstrual and hormonal status where it is appropriate, and explore whether food-based antioxidant strategies differ across sexes and life stages [68,69].

Training status and responder profiles also require closer attention. Highly trained athletes, recreationally active adults, older adults, untrained individuals, and elite master-level athletes may not respond in the same way to the same antioxidant-rich food. Elite master-level athletes may be particularly informative because they combine age-related recovery considerations with long-term training exposure and competitive performance demands. Trained individuals often have more developed endogenous antioxidant systems and may show smaller biomarker changes after food-based interventions [3,4]. Individuals with lower baseline diet quality, greater inflammation, or poorer recovery capacity may show clearer responses. Future studies should move beyond average group effects and examine responder and non-responder patterns. This would clarify whether baseline antioxidant intake, fitness level, gut microbiota, oral microbiota, age, sex, training load, and competitive status modify the effectiveness of food-based strategies [18,19,25,26,27,67].

Microbial metabolism is another priority. Many polyphenols are poorly absorbed in their parent form and require gut microbial transformation to generate smaller compounds with potential biological activity [18,19,26,27]. Nitrate-rich foods also depend partly on oral bacteria for the conversion of nitrate to nitrite, a key step in nitric oxide production [12,13,25]. These microbial pathways may help explain why some individuals respond strongly to pomegranate, berries, cocoa, green tea, or beetroot, whereas others show little change [26,27,67]. Future exercise trials should combine dietary interventions with microbial profiling, metabolomics, and measurements of circulating bioactive metabolites. Such designs would help determine how food-derived compounds are transformed and how those metabolites influence redox regulation, inflammation, vascular function, and recovery [67,76,77].

Finally, the field needs to move beyond single-biomarker interpretations. A small number of oxidative stress or inflammatory markers cannot capture the complexity of exercise-induced adaptation. Integrating metabolomics, proteomics, transcriptomics, lipidomics, and microbiomics with performance and recovery outcomes would allow researchers to examine networks of biological responses rather than isolated markers [76,77,78]. The practical goal is not to prescribe the same antioxidant strategy every day. Athletes may need different approaches depending on whether the current priority is adaptation, rapid recovery, competition readiness, immune support, or return from illness or injury [21,22,55,56]. Future research should therefore move toward a context-specific, food-based, and personalized model of redox nutrition rather than a nutrient-centered antioxidant model.

## 9. Conclusions

Exercise-induced reactive oxygen and nitrogen species have different physiological meanings depending on their magnitude, timing, and resolution. Moderate and transient redox responses can contribute to mitochondrial biogenesis, endogenous antioxidant defense, vascular regulation, inflammatory resolution, and other adaptations that support training effectiveness. When the response becomes excessive, prolonged, or poorly resolved, however, the same biological network may contribute to muscle damage, fatigue, inflammation, impaired contractile function, and delayed recovery. Therefore, antioxidant nutrition in exercise requires a more precise goal than simply lowering oxidative or nitrosative stress.

High-dose isolated antioxidant supplementation may be useful in selected settings, but routine use during adaptation-focused training should be approached cautiously. Food-based antioxidant nutrition may offer a different model. Antioxidant-rich foods deliver redox-active compounds within dietary matrices that also provide polyphenols, nitrate, fiber, minerals, vitamins, and other bioactive components. These matrices may influence recovery, vascular function, inflammatory regulation, gut-derived metabolism, and overall diet quality in ways that isolated nutrients cannot fully reproduce.

In practice, the starting point should remain a high-quality habitual diet rather than automatic antioxidant loading. Berries, tart cherry, pomegranate, cocoa, green tea, beetroot and other nitrate-rich vegetables, extra virgin olive oil, and Mediterranean-style dietary patterns may be useful when recovery demands are high and when their use matches the training phase, competition schedule, baseline diet quality, and individual response. They may be best viewed as conditional recovery tools rather than universal ergogenic aids.

Overall, food-based antioxidant nutrition is best interpreted as a context-specific strategy for supporting recovery rather than as a universal method for suppressing exercise-induced redox activity. Its practical value is likely to depend on training phase, recovery demand, dietary baseline, food matrix, dose, timing, and individual responsiveness. Future studies should test these strategies with clearer attention to intervention form, short- versus long-term outcomes, sex- and age-related differences, gut microbial metabolism, and the distinction between acute recovery and durable training adaptation.

## Figures and Tables

**Table 1:** Practical and mechanistic comparison between high-dose isolated antioxidant supplementation and food-based antioxidant strategies.

Feature	High-Dose Isolated Antioxidant Supplementation	Food-Based Antioxidant Strategies	Implications for Training Adaptation
Typical form	Capsules, tablets, powders, concentrated extracts, or single-nutrient formulations	Whole foods, beverages, meals, or dietary patterns rich in multiple bioactive compounds	Food-based strategies more closely reflect habitual dietary intake
Common examples	High-dose vitamin C, high-dose vitamin E, isolated antioxidant extracts	Berries, tart cherry, pomegranate, cocoa, green tea, beetroot, leafy greens, extra virgin olive oil, Mediterranean-style meals	Whole foods provide broader physiological support beyond antioxidant activity
Dose profile	Often high and pharmacological relative to normal food intake	Usually moderate and distributed across meals or dietary patterns	Lower likelihood of strong redox suppression when used as part of habitual diet
Nutrient complexity	Narrow compound exposure, often one or two isolated nutrients	Polyphenols, nitrate, vitamins, minerals, fiber, organic acids, unsaturated fats, and other bioactives	Multiple nutrients may act together through complementary mechanisms
Main biological emphasis	Direct antioxidant effect and reduction in oxidative biomarkers	Redox modulation, inflammatory regulation, vascular support, gut microbiota-derived metabolites, overall diet quality	Supports a shift from oxidative stress suppression to contextual redox regulation
Relationship to adaptive signaling	May attenuate selected redox-sensitive adaptations under some conditions, especially with chronic high-dose use	May support recovery while preserving adaptive signaling when matched to training context	Food-based strategies may be more compatible with long-term training adaptation
Recovery application	May be useful in selected clinical or high-stress contexts, but requires caution	Useful during heavy training, competition congestion, muscle-damaging exercise, travel, or low dietary variety	Application should be targeted rather than automatic
Diet quality contribution	Limited contribution to total diet quality, energy intake, protein, carbohydrate, fiber, or micronutrient sufficiency	Contributes to overall dietary quality and may support multiple recovery pathways	Should complement foundational sports nutrition practices
Individual variability	Response may depend on dose, baseline antioxidant status, training status, and outcome domain	Response may depend on food matrix, gut microbiota, oral microbiota, baseline diet, sex, age, and training status	Personalized and periodized application is needed
Practical caution	Avoid routine long-term high-dose use during adaptation-focused training unless clinically indicated	Avoid assuming that all antioxidant-rich foods improve performance in all contexts	The goal is not maximal antioxidant intake, but appropriate redox modulation

**Table 2:** Summary of food-based antioxidant sources, proposed mechanisms, potential exercise-related outcomes, overall strength of evidence, and evidence considerations. The effects of these foods depend on exercise context, dose, timing, food matrix, training status, baseline diet quality, and individual responsiveness. The “overall strength of evidence” column provides a narrative judgment of relative evidence maturity and should not be interpreted as a formal certainty-of-evidence or GRADE assessment.

Food-Based Source	Major Bioactive Compounds	Proposed Mechanisms	Potential Exercise-Related Outcomes	Overall Strength of Evidence	Evidence Considerations
Berries, including blueberries and blackcurrants	Anthocyanins, flavonols, phenolic acids, vitamin C, fiber	Redox modulation, inflammatory regulation, endothelial support, substrate metabolism modulation, and possible gut microbiota-derived metabolite effects	May support recovery, vascular function, substrate metabolism, inflammatory resolution, and exercise performance under selected conditions	Preliminary to moderate; evidence is growing but heterogeneous across berry type, dose, timing, and outcome domain	Evidence is growing but heterogeneous. Inconsistent findings may reflect differences in berry type, food form, anthocyanin dose, timing, exercise-induced muscle damage, training status, baseline diet quality, gut-derived phenolic metabolites, and outcome domain [20,27,33,34,35,36,37]
Tart cherry	Anthocyanins, flavonoids, phenolic acids, melatonin-related compounds	Modulation of inflammation, oxidative stress, muscle soreness, and recovery-related signaling	May reduce delayed-onset muscle soreness and support recovery of muscle function after strenuous endurance, resistance, or intermittent exercise	Relatively strong for muscle soreness and short-term functional recovery after strenuous or muscle-damaging exercise	Evidence is strongest under high muscle-damage or high-recovery-demand conditions. Routine use during all training phases may not be necessary [28,29,30,31,32]
Cocoa and dark chocolate	Flavanols, especially epicatechin and catechin	Endothelial nitric oxide support, vascular function, microvascular blood flow, and redox modulation	May support vascular responses, oxygen delivery, and selected performance or recovery outcomes	Moderate for vascular function; more preliminary or product-dependent for exercise recovery and performance outcomes	Product quality is critical. Many commercial chocolate products have low flavanol content and high sugar or fat content, limiting direct translation to practical recovery nutrition [23,38,39]
Pomegranate	Ellagitannins, anthocyanins, phenolic acids, urolithin precursors	Redox and inflammatory modulation, endothelial support, nitric oxide-related vascular effects, and gut microbial conversion to urolithins	May support strength recovery, soreness reduction, blood flow, and recovery after resistance or eccentric exercise	Moderate but heterogeneous; evidence is more developed for vascular and recovery-related outcomes than for consistent performance benefits	Responses may vary according to product form, polyphenol content, dose, timing, participant characteristics, and gut microbial metabolism [24,26,40,41]
Green tea	Catechins, especially epigallocatechin gallate, epicatechin, epigallocatechin, and epicatechin gallate	Antioxidant defense, inflammatory regulation, metabolic effects, and possible fat oxidation support	May reduce selected oxidative stress markers and contribute to long-term redox balance as part of a broader dietary pattern	Preliminary to moderate; findings vary by catechin dose, caffeine content, intervention duration, and outcome domain	Evidence is mixed. Brewed green tea should be distinguished from concentrated green tea extracts, which may provide much higher catechin exposure and require greater safety caution [42,43,44,45,46,47]
Beetroot and nitrate-rich vegetables	Dietary nitrate, betalains, polyphenols, minerals	Nitrate-nitrite-nitric oxide pathway, vasodilation, oxygen delivery, muscle efficiency, vascular support, and redox-related effects	May improve exercise efficiency, endurance performance, vascular function, and selected high-intensity exercise outcomes	Relatively strong for nitric oxide-related vascular function and exercise efficiency; more mixed for recovery-specific outcomes	Responses depend on nitrate dose, timing, training status, oral nitrate-reducing bacteria, exercise modality, product form, and processing method. Beetroot effects should not be attributed to nitrate alone because betalains, polyphenols, minerals, and matrix-related factors may also contribute to physiological responses [12,13,25,48,49,50]
Extra virgin olive oil	Monounsaturated fatty acids, hydroxytyrosol, tyrosol, oleuropein derivatives, oleocanthal, and other olive-derived phenolics	Anti-inflammatory effects, endothelial support, redox modulation, cardiometabolic support, and possible recovery-related effects	May contribute to long-term recovery environment, vascular health, inflammatory balance, and overall dietary quality	Preliminary to moderate; evidence is stronger for cardiometabolic and inflammatory contexts than for direct exercise recovery outcomes	Evidence in athletes remains less direct than for tart cherry or beetroot. Extra virgin olive oil is best interpreted as part of a long-term Mediterranean-style dietary pattern rather than as an acute recovery supplement [51,52,53,54]
Mediterranean-style dietary patterns	Fruits, vegetables, legumes, whole grains, nuts, seeds, fish, herbs, extra virgin olive oil, polyphenols, fiber, and unsaturated fats	Whole-diet anti-inflammatory effects, redox balance, cardiometabolic support, micronutrient sufficiency, gut health, and dietary quality enhancement	May support long-term health, recovery capacity, immune function, vascular function, and training sustainability	Moderate for overall diet quality, inflammatory and vascular health; indirect for acute exercise recovery and training adaptation	Must be adapted to sport-specific energy, carbohydrate, protein, fluid, electrolyte, and competition demands [21,51,55,56]

**Table 3:** Practical decision guide for periodized food-based antioxidant strategies and selected concentrated antioxidant products. This table is intended as a conceptual, evidence-informed decision guide; several recommendations rely on indirect evidence, mechanistic reasoning, or extrapolation from related populations rather than intervention trials conducted specifically in each listed population or context.

Exercise Context	Primary Physiological Challenge	Food-Based Strategy	Proposed Mechanism	Practical Caution	Representative Evidence
Adaptation-focused endurance training	Need to preserve mitochondrial and redox-sensitive signaling	Maintain a diverse diet rich in fruits, vegetables, whole grains, legumes, nuts, and seeds	Supports baseline redox homeostasis and micronutrient sufficiency without aggressive redox suppression	Avoid routine long-term high-dose isolated vitamin C or vitamin E supplementation unless clinically indicated	[3,4,14,15,16,17,21,55,56,74,75]
Heavy training block	Accumulated fatigue, inflammation, muscle soreness, and incomplete recovery	Increase polyphenol-rich foods such as berries, tart cherry, pomegranate, and colorful fruits	May help regulate inflammation, oxidative stress, and muscle soreness while supporting training continuity	Use as recovery support, not as a substitute for load management, energy intake, carbohydrate, protein, and sleep	[6,7,8,20,28,29,30,31,32,33,34,35,36,37,40,58]
Congested competition period	Rapid recovery between repeated events	Tart cherry, pomegranate, nitrate-rich vegetables, fruit-based recovery meals, and antioxidant-rich snacks	May reduce soreness, support vascular function, and help maintain readiness between events	Short-term targeted use is more appropriate than assuming constant daily use is needed Short-term concentrated antioxidant products or selected supplements may be considered when recovery windows between successive competitions are brief	[28,32,40,48,49,57,58,61,62,63,74,75]
Resistance or eccentric exercise block	Muscle damage, delayed-onset muscle soreness, and temporary strength loss	Tart cherry, berries, pomegranate, and protein-rich meals with colorful fruits or vegetables	May support inflammatory resolution, redox balance, and muscle repair when combined with adequate protein	Avoid excessive antioxidant dosing when hypertrophy or strength adaptation is the main goal	[6,7,8,28,29,30,31,32,33,34,35,36,37,40,59]
High-intensity intermittent or team-sport training	Repeated sprint stress, neuromuscular fatigue, inflammation, and limited recovery time	Mixed meals with fruits, vegetables, nitrate-rich foods, cocoa, and adequate carbohydrate	Supports glycogen restoration, vascular function, redox balance, and immune regulation	Antioxidant foods should complement, not replace, carbohydrate recovery and hydration strategies	[21,28,32,48,49,50,57,58,59,60,61,62,63]
Heat stress or environmental strain	Dehydration, electrolyte loss, increased cardiovascular strain, and oxidative stress	Fluids, electrolytes, fruits, vegetables, and polyphenol-rich foods with high water content	Supports hydration, electrolyte balance, vascular function, thermoregulation, and redox regulation	Antioxidant intake alone cannot compensate for inadequate fluid replacement, electrolyte replacement, heat mitigation, or cooling strategies	[21,22,60,71,72,73]
Older adults undertaking exercise training	Chronic low-grade inflammation, anabolic resistance, slower recovery, and vascular dysfunction	Mediterranean-style diet, protein plus polyphenol-rich foods, nitrate-rich vegetables, and extra virgin olive oil	Supports muscle remodeling, vascular health, inflammatory balance, and overall diet quality	Ensure adequate total energy, protein, vitamin D, and resistance training stimulus Concentrated antioxidant products should be considered cautiously and only when heavier training periods or recovery limitations justify short-term support	[25,51,59,64,65,66]
Low dietary quality or restricted food variety	Insufficient micronutrients, low polyphenol intake, and impaired recovery environment	Improve habitual intake of fruits, vegetables, legumes, nuts, seeds, tea, and whole grains	Enhances total diet quality, antioxidant defense, gut health, and recovery capacity	Correcting the overall diet should be prioritized before adding supplements	[18,19,20,21,22,51,74,75]
Travel or tournament settings	Irregular meals, sleep disruption, inflammation, and gastrointestinal challenges	Portable food-based options such as berries, tart cherry products, fruit, nuts, cocoa-based foods, and nitrate-rich vegetable products	Provides practical recovery support and maintains diet quality under logistical constraints	Choose familiar foods to reduce gastrointestinal risk before competition; supplements or sports foods may be useful when food access is limited Concentrated products or selected supplements may be considered when familiar antioxidant-rich foods are unavailable or poorly tolerated	[21,22,28,32,40,57,58,61,62,63,74,75]
Return from illness or high cumulative fatigue	Immune strain, persistent fatigue, and impaired training tolerance	Mediterranean-style meals, colorful fruits and vegetables, protein-rich foods, and adequate fluids	Supports immune regulation, inflammatory resolution, and gradual restoration of training capacity	Training load reduction and medical evaluation may be needed if fatigue persists During early recovery from illness or injury, concentrated products should be individualized and used cautiously, particularly when appetite, food tolerance, or dietary variety is limited	[21,22,25,51,58,59,64,65,74,75]

## Data Availability

Not applicable.

## Supplementary Materials

The following supporting information can be downloaded at: https://www.mdpi.com/article/10.3390/nu18132115/s1, Table S1: Structured narrative literature search framework, evidence identification concepts, and representative search strings.

## Abbreviations

The following abbreviations are used in this manuscript:

AMP	Adenosine monophosphate
AMPK	AMP-activated protein kinase
ATP	Adenosine triphosphate
DNA	Deoxyribonucleic acid
NADPH	Nicotinamide adenine dinucleotide phosphate
NO	Nitric oxide
Nrf2	Nuclear factor erythroid 2-related factor 2
PGC-1α	Peroxisome proliferator-activated receptor gamma coactivator 1-alpha
RONS	Reactive oxygen and nitrogen species
ROS	Reactive oxygen species
RNS	Reactive nitrogen species
