# Supplementary material for: Food-Based Antioxidant Nutrition for Exercise Recovery and Training Adaptation: A Narrative Review and Conceptual Framework for Redox Signaling, Dietary Matrices, and Periodized Application
Source: Nutrients. 2026 Jun 29;18(13):2115. doi: 10.3390/nu18132115 (PMC13363514; doi:10.3390/nu18132115)
Supplement: Supplementary file 1 [file nutrients-18-02115-s001.zip › nutrients-4368538-supplementary.pdf]

Supplementary Table S1. Structured narrative literature search framework, evidence identification concepts, and representative search strings

Framework domain	Search focus	Examples of search terms	Evidence types and prioritization	Role in the narrative synthesis
Exercise and adaptation	Exercise training, recovery, performance, muscle damage, and training adaptation	exercise; exercise training; recovery; exercise recovery; muscle damage; delayed-onset muscle soreness; training adaptation; endurance exercise; resistance exercise; high-intensity exercise; team-sport exercise	Human exercise and sports nutrition studies were prioritized when practical recommendations were discussed. Experimental and applied studies were used to define exercise contexts and recovery outcomes.	Defined the exercise context and outcomes relevant to recovery, performance readiness, and training adaptation.
Redox biology	ROS/RNS production, oxidative stress, nitrosative stress, redox homeostasis, and redox-sensitive signaling	oxidative stress; nitrosative stress; reactive oxygen species; ROS; reactive nitrogen species; RNS; redox homeostasis; redox signaling; Nrf2; AMPK; PGC-1α; nitric oxide; mitochondrial biogenesis	Mechanistic, cellular, animal, and human studies were considered when they contributed biological plausibility. Human exercise evidence was prioritized when available.	Supported interpretation of the dual role of exercise-induced ROS/RNS as adaptive signals and potential contributors to oxidative or nitrosative damage.
Antioxidant nutrition	Dietary antioxidants, isolated	antioxidants; dietary antioxidants;	Reviews, randomized trials, crossover studies,	Distinguished high-dose isolated antioxidant

Framework domain	Search focus	Examples of search terms	Evidence types and prioritization	Role in the narrative synthesis
	antioxidant supplementation, polyphenols, and food-based nutrition	antioxidant supplementation; vitamin C; vitamin E; polyphenols; flavonoids; anthocyanins; catechins; food-based nutrition; whole foods; dietary patterns	meta-analyses, and consensus or position statements were prioritized when available. High-dose isolated supplementation studies were used mainly to distinguish supplement-based effects from food-based approaches.	supplementation from food-based antioxidant strategies and supported the conceptual shift from antioxidant suppression to redox modulation.
Food-based sources	Polyphenol-rich foods, nitrate-rich foods, extra virgin olive oil, and dietary-pattern approaches	tart cherry; berries; blueberries; blackcurrant; pomegranate; cocoa; green tea; beetroot; dietary nitrate; nitrate-rich vegetables; leafy greens; extra virgin olive oil; Mediterranean diet	Human studies were prioritized for food-based recovery and performance outcomes. Mechanistic studies were used when relevant to bioavailability, vascular function, gut microbial metabolism, or food matrix effects.	Identified food-based sources relevant to recovery, inflammation, vascular function, gut-derived metabolites, redox balance, and overall diet quality.
Food matrix, bioavailability, and microbial metabolism	Dietary matrices, absorption, metabolism, gut microbiota, oral microbiota, and metabolite production	food matrix; dietary matrix; bioavailability; bioefficacy; gut microbiota; gut microbiome; oral microbiota; nitrate-reducing	Mechanistic and translational studies were included to explain inter-individual variability and biological plausibility.	Explained why whole-food strategies may differ from isolated supplements and why responses may depend on

Framework domain	Search focus	Examples of search terms	Evidence types and prioritization	Role in the narrative synthesis
		bacteria; phenolic metabolites; urolithins; nitric oxide bioavailability	Studies on gut-derived metabolites and nitrate metabolism were used to interpret responder variability.	food form, processing, microbial metabolism, and individual responsiveness.
Practical application and periodization	Training phase, recovery demand, competition schedule, diet quality, and sports nutrition practice	sports nutrition; recovery nutrition; periodized nutrition; nutrition periodization; training phase; heavy training; congested competition; travel; heat stress; dietary pattern; energy availability; carbohydrate; protein; hydration	Sports nutrition consensus statements, applied reviews, and human studies were prioritized for practical interpretation. Evidence was interpreted cautiously where direct athlete-specific data were limited.	Guided the development of context-specific recommendations for applying food-based antioxidant strategies across adaptation-focused training, heavy training blocks, competition periods, and recovery phases.
Eligibility and exclusion logic	Relevance to exercise, nutrition, redox biology, recovery, and food-based antioxidant strategies	Not applicable as a keyword domain	Sources were not prioritized when they were unrelated to exercise training or recovery, focused only on disease treatment without clear	Clarified the selection logic used to reduce conceptual drift and improve transparency in the narrative synthesis.

Framework domain	Search focus	Examples of search terms	Evidence types and prioritization	Role in the narrative synthesis
			exercise or nutritional relevance, did not address antioxidant or redox-related mechanisms, or were editorials, opinion pieces, conference abstracts, or non-peer-reviewed materials. English-language peer-reviewed publications were prioritized; however, non-English articles were considered when they appeared directly relevant, had sufficient information available through an English abstract or reliable translation, and contributed important mechanistic or	

Framework domain	Search focus	Examples of search terms	Evidence types and prioritization	Role in the narrative synthesis
			applied evidence.	

Table note: This table summarizes the structured search concepts used to support the narrative synthesis. Representative search strings are provided below to improve transparency. It is not intended to represent a fully reproducible systematic review search strategy and was used to guide conceptual synthesis rather than pooled inference or formal certainty-of-evidence grading.

Representative search strings

Representative search strings used to guide the structured narrative literature search included the following examples:

1. (“exercise” OR “exercise training” OR “endurance training” OR “resistance training”) AND (“reactive oxygen species” OR “reactive nitrogen species” OR “oxidative stress” OR “redox signaling” OR “redox homeostasis”) AND (“training adaptation” OR “mitochondrial biogenesis” OR “Nrf2” OR “AMPK” OR “PGC-1α”)

2. (“exercise recovery” OR “muscle damage” OR “delayed onset muscle soreness” OR “inflammation” OR “fatigue”) AND (“dietary antioxidants” OR “antioxidant nutrition” OR “polyphenols” OR “anthocyanins” OR “food-based nutrition”)

3. (“tart cherry” OR “berries” OR “blueberry” OR “blackcurrant” OR “pomegranate” OR “cocoa” OR “green tea”) AND (“exercise” OR “sports nutrition” OR “recovery” OR “muscle soreness” OR “oxidative stress” OR “inflammation”)

4. (“beetroot” OR “dietary nitrate” OR “nitrate-rich vegetables” OR “nitric oxide”) AND (“exercise performance” OR “vascular function” OR “endothelial function”

OR “exercise efficiency” OR “recovery”)

5. (“Mediterranean diet” OR “extra virgin olive oil” OR “dietary pattern” OR “whole foods” OR “food matrix”) AND (“oxidative stress” OR “inflammation” OR “vascular function” OR “gut microbiota” OR “exercise recovery”)
